# What the radiologist should know about the role of percutaneous gastrostomy: a pictorial essay

**DOI:** 10.1590/0100-3984.2021.0066-en

**Published:** 2022

**Authors:** Tiago Kojun Tibana, Leonardo Verza, Bernardo Caetano da Silva Rodrigues, Lucas Moretti Monsignore, Daniel Giansante Abud, Thiago Franchi Nunes

**Affiliations:** 1 Department of Vascular and Interventional Radiology, Universidade Federal de Mato Grosso do Sul (UFMS), Campo Grande, MS, Brazil.; 2 Department of Imaging, A.C.Camargo Cancer Center, São Paulo, SP, Brazil.; 3 Department of Interventional Radiology, Hospital Federal Ipanema (HFI), Rio de Janeiro, RJ, Brazil.; 4 Division de of Interventional Radiology, Department of Radiology, Faculdade de Medicina de Ribeirão Preto da Universidade de São Paulo (FMRP-USP), Ribeirão Preto, SP, Brazil.

**Keywords:** Gastrostomy, Radiology, interventional, Fluoroscopy, Tomography, X-ray computed, Gastrostomia, Radiologia intervencionista, Fluoroscopia, Tomografia computadorizada

## Abstract

The image-guided gastrostomy techniques, as transoral and transabdominal, can be performed when there is a failure of the endoscopic procedure or in some specific clinical scenarios. This pictorial essay intends to show the percutaneous gastrostomy techniques, indications, technical approaches, post-procedure care, and complications.

## Abstract (pt)

As técnicas de gastrostomia guiadas por imagem, por via transoral e transabdominal podem ser realizadas quando há falha na técnica endoscópica ou em cenários clínicos em que a endoscopia não pode ser realizada. Este ensaio iconográfico pretende mostrar as técnicas de gastrostomia percutânea, suas indicações, aspectos técnicos, cuidados pós-procedimento e complicações.

## INTRODUCTION

Gastrostomy has been performed since the 19th century. However, when it is performed by open surgery, it requires general anesthesia, which is associated with high morbidity and mortality. Therefore, open gastrostomy has been replaced by percutaneous methods. In 1980, Gauderer et al.^(1)^ described the first successful percutaneous gastrostomy, which was achieved with the aid of endoscopy. A year later, Preshaw^(2)^ described the first successful percutaneous gastrostomy using fluoroscopic guidance, which has become established as a safe, effective technique for enteral nutrition or gastric decompression.

Image-guided gastrostomy, whether transoral^(3)^ or transabdominal^(4)^, can be performed when the endoscopic technique fails or in clinical settings where endoscopy cannot be performed, including rigid stenosis of the upper gastrointestinal tract, hernia of large hiatus, or significant obesity^(3-5)^. Consistently high (95-100%) success rates have been reported for transabdominal gastrostomy^(6,7)^. In a meta-analysis, Wollman et al.^(8)^ found that the success rate for transoral gastrostomy is 99.2%, compared with 95.7% for the transabdominal endoscopic technique.

The main disadvantage of an exclusively percutaneous method is the smaller-diameter tubes that are typically used, which are more prone to occlusion. In addition, fluoroscopic guidance may be required when those tubes have to be replaced^(9)^.

Since the introduction of percutaneous gastrostomy in 1980, various procedures have been developed. The techniques and the equipment used in percutaneous insertion were improved and are now considered to represent one of the best options for gastrostomy^(9)^.

## INDICATIONS

To be considered candidates for gastrostomy, patients should be at high risk for malnutrition and not likely to recover their ability to eat in the short term or should require long-term gastric decompression. Indications include the following^(4,9,10)^: neurogenic dysphagia with high risk of aspiration; a history of stroke, cognitive impairment, or reduced level of consciousness; degenerative syndromes; head and neck cancer; a history of oral or throat surgery; syndromes that progress to malabsorption; Crohn’s disease; the need for nutritional supplementation; systemic sclerosis; radiation enteritis; severe burns; and profound depression.

## CONTRAINDICATIONS

The relative contraindications to gastrostomy include ascites, partial gastrectomy, large hiatal hernia, gastric volvulus, esophagectomy with gastric traction, colonic interposition, diaphragmatic denervation with upward displacement of the stomach, prolonged use of steroids, and immunosuppression. The main absolute contraindications are uncorrectable coagulopathies, active peritonitis, mesenteric ischemia, obstruction of the gastrointestinal tract (unless the indication is decompression), and portal hypertension with gastric varices, which can bleed profusely^(4,9,10)^.

## INTERVENTIONAL TECHNIQUES

### Retrograde transabdominal gastrostomy

**Figure 1:**
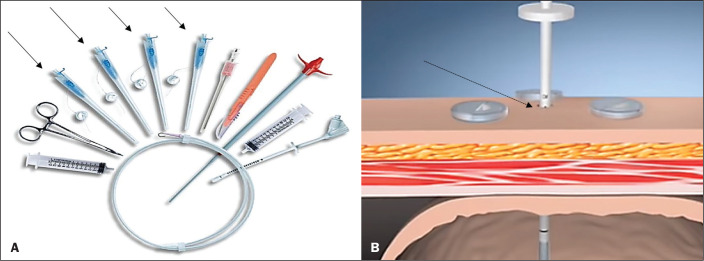
In A, a kit used for transabdominal insertion with fasteners (arrows). In B, schematic demonstrating the puncture (arrow) between the fasteners.

**Figure 2:**
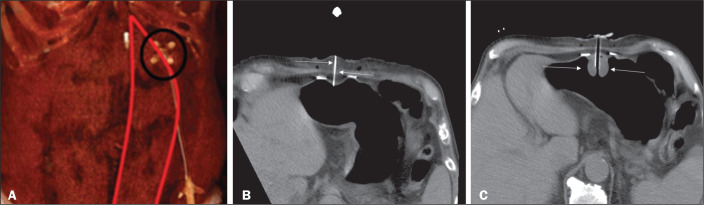
CT-guided retrograde gastrostomy. In A, gastropexy. In B, puncture between the fasteners (arrows). In C, final appearance with an inflated cuff (arrows) inside the stomach.

**Figure 3:**
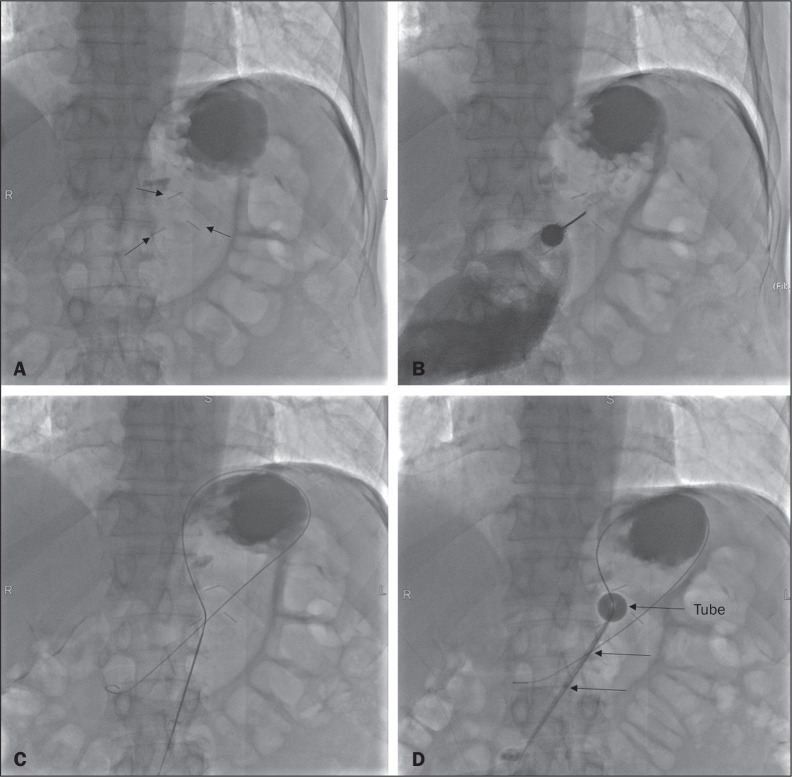
Fluoroscopy-guided retrograde gastrostomy. In A, gastropexy (arrows). In B, puncture between the fasteners. In C, guidewire inside the stomach. In D, final appearance with the tube in place (arrows).

A specific kit is typically used for transabdominal gastrostomy^(4)^, as depicted in Figure 1. After insufflation of the stomach with air or oxygen via a nasogastric tube, anteroposterior and lateral X-ray views of the stomach are obtained in order to confirm interposition of the transverse colon. A needle is introduced through the anterior abdominal wall into the stomach and contrast is injected to confirm opacification of the gastric lumen. Two or more gastropexy T-fasteners are advanced and implanted into the gastric body under guidance by fluoroscopy or computed tomography (CT), to secure the anterior wall of the stomach to the abdominal wall, and the puncture is then performed between the fasteners. The transabdominal path is serially dilated guided by a 0.035-in. guidewire (Amplatz; Cook Medical, Bloomington, IN, USA), and a 20F detachable sheath is advanced into the gastric lumen. The balloon gastrostomy tube is then advanced over the guidewire, inflated with a small volume of diluted contrast solution, retracted into the anterior gastric wall, and secured in place by an retention disc advanced to the overlying skin. The correct positioning is confirmed by opacification of gastric lumen after contrast injection (Figures 2 and 3).

In the CT-guided technique, the gastric antrum can be insufflated with a gastric tube or by direct puncture of the empty stomach with a Chiba 21G needle and subsequent distention with room air. Two or three fasteners are inserted to fix the gastric body to the abdominal wall. A needle from the guidewire kit is inserted between the fasteners, and sequential detachable dilators are inserted through the needle track. The balloon gastrostomy tube is then advanced over the guidewire. The retention balloon is inflated, and the tube is partially retracted for adequate fixation to the anterior gastric wall. All steps of the procedure and the final position of the probe are confirmed by CT^(11)^, as illustrated in Figure 2. In brief, the steps are as follows: guided puncture; insertion of fasteners; puncture between fasteners; dilation of the path along the guidewire; detachable sheath insertion; passage of the tube over the guidewire; and balloon inflation.

### Antegrade transoral techniques

**Figure 4:**
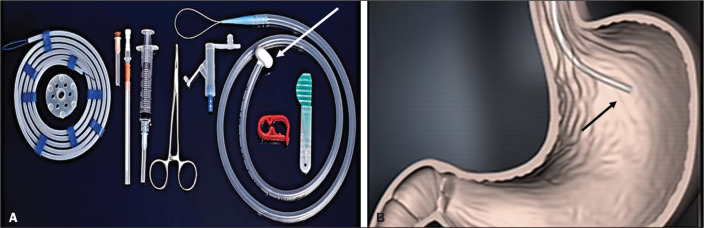
In A, percutaneous endoscopic gastrostomy system used in fastener-free antegrade techniques with a mushroom-tipped tube (arrow). In B, position of the sheath (arrow) within the stomach.

**Figure 5:**
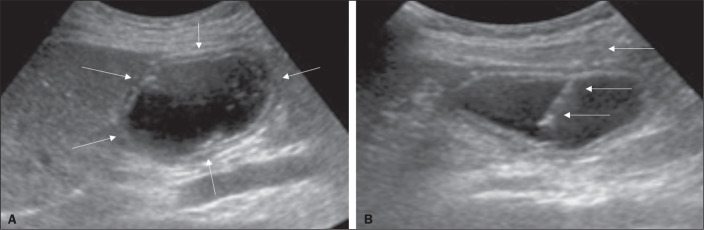
Gastric distention with saline solution (arrows in A) and ultrasound-guided puncture (arrows in B).

In one antegrade transoral gastrostomy technique, the tube is placed by an antegrade approach using an percutaneous endoscopic gastrostomy system^(9)^, as depicted in Figure 4. The stomach is insufflated with air or oxygen via a nasogastric tube. Anteroposterior and lateral X-rays are then obtained, after which the puncture is performed with a 21G or 19G needle through the anterior abdominal wall to the stomach between the body and the antrum. In another technique, saline solution is infused through a nasogastric tube and ultrasound-guided puncture is performed (Figure 5).

**Figure 6:**
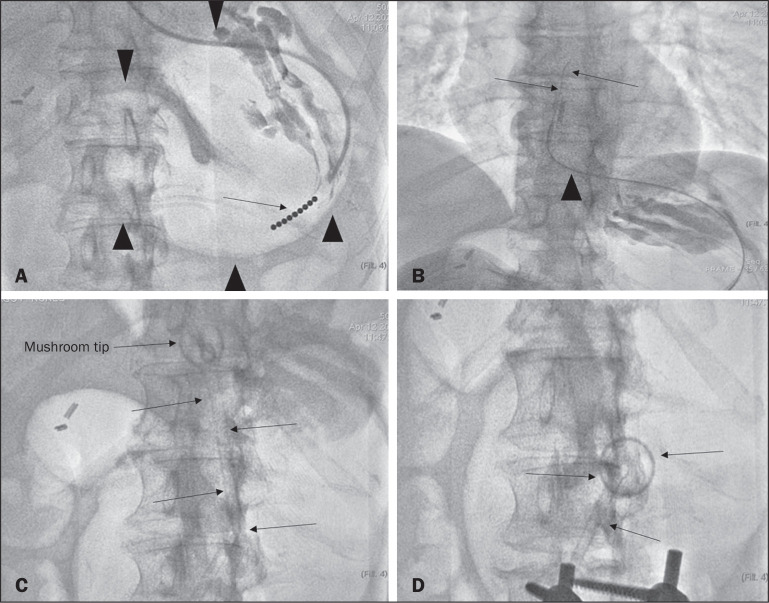
Fluoroscopy-guided antegrade gastrostomy. In A, contrast infusion through the nasogastric tube (arrows) and stomach insufflation (arrowheads). In B, guidewire (arrows) and catheter (arrowhead) along the path from the stomach to the esophagus and to the mouth. In C, gastrostomy tube being positioned (arrows). In D, final positioning of the gastrostomy tube (arrows).

In the first technique, the needle is replaced with a guidewire and a 6F angled diagnostic catheter. A 0.035-in. hydrophilic guidewire is inserted into the catheter, is routed into the esophagus, and finally emerges from the mouth (Figure 6). A double guidewire is passed through the catheter and fixed by the tie onto the tube with a “mushroom” tip. The gastrostomy tube is then inserted into the mouth, through the esophagus, and finally into the stomach so that the retention “mushroom” is securely positioned against the anterior wall of the stomach. In brief, the steps are as follows: guided puncture; replacement of the needle with a diagnostic catheter and hydrophilic guidewire; passage of the guidewire and catheter through the esophagus to the mouth; replacement of the hydrophilic guidewire with the double guidewire; fixation of the double guidewire in the mushroom-retained tube; and insertion/placement of the tube into the stomach via the mouth and esophagus.

**Figure 7:**
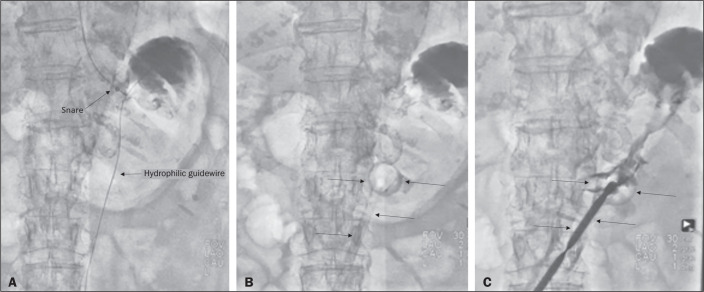
Fluoroscopy-guided antegrade gastrostomy. In A, capture of the hydrophilic guidewire by the snare. In B and C, the final appearance, with contrast injection through the tube (arrows).

In the second technique (Figure 7), the needle is replaced with a 6F angiographic introducer. A 0.035-in hydrophilic guidewire and diagnostic catheter are then introduced into the stomach. The hydrophilic guidewire and catheter are then captured from within the stomach, by a snare inside a 7F or 8F sheath, and pulled up to the mouth. The hydrophilic guidewire is replaced with a double guidewire, which is then fixed by the tie onto a tube with a mushroom tip. The gastrostomy tube is then inserted into the mouth, through the esophagus, and finally into the stomach. The correct positioning of the tube is confirmed by opacification of gastric lumen after contrast injection. In brief, the steps are as follows: guided puncture; replacement of the needle with an angiographic introducer; passage of a diagnostic catheter and hydrophilic guidewire into the stomach; passage of the sheath through the mouth to the stomach; passing snare inside the sheath; capture of the hydrophilic guidewire and catheter through the snare, which are then pulled to the mouth; fixation of the double guidewire in the mushroom-retained tube; and insertion/placement of the tube into the stomach via the mouth and esophagus.

## MEDICATIONS AND PERIPROCEDURAL CARE

For patients undergoing gastrostomy, anesthesia is required, usually involving conscious sedation and local nerve blocks. It is crucial to have a multidisciplinary team for intravenous drug administration and patient monitoring. Midazolam and fentanyl are usually sufficient, although should be monitored continuously during the procedure. Intraluminal distention is fundamental and essential for the success of the procedure. Antispasmodics such as hyoscine butylbromide are useful, particularly in cases in which it is difficult to maintain gastric distention.

## POSTPROCEDURAL PROTOCOLS

Postprocedural imaging is rarely necessary. However, if there is any question about the intragastric placement, unenhanced CT can be performed for clarification. Chest X-rays are of no value to exclude perforation, because pneumoperitoneum is a natural consequence of puncture of a distended stomach and free subdiaphragmatic air should not be considered a reliable sign of intestinal perforation. There is no consensus on when feeding can be started after tube insertion. Most centers apply a 4-6 hour fasting period before testing the tube by injecting water. Prior to receiving enteral nutrition, the patient should be evaluated by a trained member of the nutritional support team to decide whether the tube can be used.

## COMPLICATIONS

Major complications of gastrostomy include hemorrhage, peritonitis, colon perforation, and severe skin infection, although the incidence rates of such complications are low (< 2.0%). Minor complications are more common and include peristomal infection, leaks, tube occlusion, and tube displacement, with incidence rates ranging from 1.3% to 45%^(4,9,10)^.

## CONCLUSION

Percutaneous gastrostomy is a safe, effective procedure, with good success rates and low complication rates, being feasible when conventional techniques cannot be performed. The main limitation of percutaneous gastrostomy is the smaller tube size, which can lead to a higher obstruction rate^(4,6,8-11)^.
